# Formulation and Optimization of Oral Mucoadhesive Patches of Myrtus Communis by Box Behnken Design

**DOI:** 10.15171/apb.2017.053

**Published:** 2017-09-25

**Authors:** Mahbubeh Hashemi, Vahid Ramezani, Mohammad Seyedabadi, Ali Mohamad Ranjbar, Hossein Jafari, Mina Honarvar, Hamed Fanaei

**Affiliations:** ^1^Department of Pharmaceutics, Faculty of Pharmacy, Shahid Sadoughi University of Medical Sciences, Yazd, Iran.; ^2^Department of Pharmacology, School of Medicine, Bushehr University of Medical Sciences, Bushehr, Iran.; ^3^Department of pharmacognosy, Faculty of Pharmacy, Shahid Sadoughi University of Medical Sciences, Yazd, Iran.; ^4^Department of Physiology, School of Medicine, Zahedan University of Medical Sciences, Zahedan, Iran.

**Keywords:** Myrtus communis, Oral patch, Methyl cellulose, Gelatin, Polyvinyl pyrrolidone, Pectin

## Abstract

***Purpose:*** Recurrent aphthous stomatitis (RAS) is the most common painful ulcerative disease of oral mucosa happening in ~20% of people. Aimed to develop Myrtus communis L. (Myrtle) containing oral patches, we applied box-behnken design to evaluate the effect of polymers such as Polyvinyl pyrrolidone (PVP), Gelatin, Methylcellulose (MC) and Pectin.

***Methods:*** The patches properties such as tensile strength, folding endurance, swelling index, thickness, mucoadhesive strength and the pattern of myrtle release were evaluated as dependent variables. Then, the model was adjusted according to the best fitted equation with box behnken design.

***Results:*** The results indicated that preparation of myrtle patch with hydrophilic polymers showed the disintegration time up to 24h and more. Using of polyvinyl pyrrolidone as a water soluble polymer and a pore-former polymer led to faster release of soluble materials from the patch to 29 (min^-1^). Also it decreases swelling index by increasing the patch disintegration. Gelatin and Pectin, with rigid matrix and water interaction properties, decreased the swelling ratio. Pectin increased the tensile strength, but gelatin produced an opposite effect. Thinner Myrtle patch (about 28µm) was obtained by formulation of methyl cellulose with equal ratio with polyvinyl pyrrolidone or gelatin.

***Conclusion:*** Altogether, the analysis showed that the optimal formulation was achieved with of 35.04 mg of Gelatin, 7.22 mg of Pectin, 7.20 mg of polyvinyl pyrrolidone, 50.52 mg of methyl cellulose and 20 mg of Myrtle extract.

## Introduction

Recurrent aphthous stomatitis (RAS) refers to recurring ovoid or round ulcers with yellow base and erythema in the surrounding tissue.^1^ It is the most common painful ulcerative disease of oral mucosa happening in about 20% of people.^2,3^ Even though the exact pathology is yet to be elucidated, several local, immunological and systemic factors play a role in RAS.^4-8^ As such, RAS may be an adverse effect of some medications.^9^ Clinically, RAS is classified to three classes of minor, major, and herpetiform ulcers. Having less than 5mm diameter, minor RAS is the most common type, and happens in 80% of people. Whereas, the major form usually has a diameter of more than 1cm.^10,11^Treatment protocol depends on RAS type and symptoms. In this regard, the minor ulcers will relieve after applying topical NSAIDs (Nonsteroidal anti-inflammatory drug), corticosteroids or local anesthetics.^12,13^

Myrtle is a perennial shrub widely distributed in the north of Iran. For many years, in Persian traditional medicine, Myrtle is known by its anti-hyperglycemic, antibacterial, anti-oxidant, anti-inflammatory and analgesic properties.^14-17^ These properties suggest potential efficacy of Myrtle in RAS treatment.^18^ Indeed, Myrtle paste is proven to be effective in decreasing size of ulcer, pain, severity and erythema.^19^

Mucoadhesive drug delivery systems are preferred dosage forms for treatment of RAS ulcers because; the drug is targeted to a specific region and maintained there for a long period of time. Oral patches, in particular, are more favorable because of their ability to localize the drug and its effects.^20,21^ These formulations need to be resistant enough to maintain the integrity of drug delivery during jaw movement, and have to be flexible enough to avoid the interference with normal oral activity.^22,23^

Several factors ought to be optimized for an appropriate oral patch. In this regard, experimental design is a statistical method providing the possibility of evaluation of several independent variables on a specific response with minimum number of conducted experiments. In particular, the effect of each independent variable is initially studied on a specific response. Subsequently, multiple regressions are performed to find the magnitude of effect (β) of each variable with respect to others. Finally, the interaction of independent variable was investigated to find possible synergy or antagonism. This method has been widely applied in numerous investigations to characterize and develop patch formulations.^24,25^

In the current study, we aimed to optimize a patch formulation for Myrtle drug delivery by applying box-behnken design.

## Materials and Methods

Myrtle leaves were purchased from herbal medicine market, Yazd, Iran in January, 2015. The samples were authenticated at faculty of pharmacy, Shahid Sadoughi University of Medical Sciences, Yazd, Iran. Methyl cellulose (MC, M_W_: 658.73 g/mole), Gelatin (M_W_: 180.16 g/mole), Pectin (PE, M_W_: 194.14 g/mole), Polyvinyl pyrrolidone (PVP, M_W_: 112.89 g/mole), Propylene glycol (PG) and Folin-Ciocalteu reagent were purchased from Sigma (USA). All reagents were of analytical grade.

The leaves were powdered and then extraction was performed via percolation by Ethanol, 80% (v/v) at room temperature. Extraction was continued until no residual ingredient was observed in TLC (thin layer chromatography) followed by UV (ultra-violent) detection. Finally, the extracts were dried under vacuum evaporator and weighted to calculate the extractable material. Extraction efficiency was 12.5 percent.

### Determination of total phenol

Total phenolic content of myrtle extract was determined in accordance with Folin–Ciocalteu method.^26^ Briefly, 1.5 mL of Folin–Ciocalteu solution (diluted 10 times) was added to 200 μL of the diluted extract or gallic acid. They were mixed completely and placed at 22°C in water bath for 5 minutes. Then, 1.5 ml of sodium carbonate (60 g.L^-1^) was added to test tubes, vortexed, and incubated at 22°C in water bath for 90 minutes. Subsequently, absorption at 725 nm was determined using a UV-visible spectrophotometer (UNICO, 4802 double beam, Dayton, NJ, USA). Finally, the total phenolic content of the extracts were determined using a calibration curve with different concentrations of gallic acid (25, 50, 75, 100, 125, 150 μg.mL^-1^).

### Preparation of muco-adhesive oral patches of Myrtle

Different formulation of myrtle oral patches were prepared by solvent casting method.^27^ According to the Table 1, the proper amounts of each polymer was dispersed in deionized water and mixed for 24 h. Then, 20 mg of myrtle extract was diluted in 1ml of water, added to other ingredient, and mixed. Subsequently, proper amounts of PG, as plasticizer, were added to above mixture, and homogenized for 2 h. The total mixture was, then, degassed using a desiccator connected with vacuum pump for 6h. Finally, each formulation was casted in petri dish (diameter of 35 mm), and kept for 48h to dry.

**Table 1:** The high and low levels of independent variables

	Independent variables	-1 Level	+1 Level
A	Gelatin	1	25
B	Pectin	5	25
C	Polyvinyl pyrrolidone	5	25
D	Methylcellulose	25	75

### Surface pH

Surface pH of the myrtle patches was determined with agar plate as described by Bottenberg *et al.*^28^ Briefly, agar solution 2% (w/v) was prepared by mixing the required agar in water and then dispersed in simulated saliva (pH: 6.2). After solidification of agar and simulated salvia, the patches were placed on the surface of agar plate and allowed to swell for 2h. The surface pH finally determined by pH indicator strip. All measurements were carried out in triplicate.

### Thickness, folding endurance and tensile strength 

Screw gauge (model: 3D CAD) was employed to obtain the thickness of patches with a precision of 0.01 mm. The thickness was measured at different points and the average value was recorded.^10^

The folding endurance of patches was evaluated with continuous folding/re-folding episodes. According to the previous investigations, the suitable patch is defined as the one enduring more than 200 sequential folding/refolding challenges.^29^

The consistency of patches was evaluated using a tensilometer. In brief, a 2×1cm strip of patch was attached to two jaws; one of the jaws was fixed and the other was moving. The tensile strength was determined as resistance against breaking apart when the strip was pulled with increments of weight in moving jaw. The dial number was recorded as the tensile strength of the patch with unit of N/cm.

### Swelling study

The swelling ratio was measured by placing the patches on the surface of 2% agar plates. Agar plates were, then, incubated at 37°C, and the patches were weighed at 5, 10, 60, and 120 min after incubation. Finally, the swelling index was calculated as: Swelling index (%) = [(W_t_ – W_0_) / W_0_] ×100; Where, W_0_ is the initial weight, and W_t_ is the weight of swelled patch after the incubation for time t.

### Ex vivo mucoadhesive strength

The mucoadhesive strength of films were investigated using a modified physically balanced instrument described by Gupta.^30^ In brief, the patch was placed between two parallel surfaces covered with buccal mucosa. The film was allowed for a while to stick to the buccal surfaces. While one of the surfaces remained constant, the other moved in response to increments of weight applied. Accordingly, the maximum required force to detach the buccal surfaces was measured by an accurate digital dynamometer. The buccal area was 2.4 cm^2^ and muco-adhesive strength of the films was reported in N/cm^2^scale.

### In vitro release study

USP apparatus Type-2 rotating paddle (Erweka, Germany) was used to evaluate the release of myrtle extract from patches. Dissolution was performed in 500 mL of phosphate buffer (pH: 6.8) at 37±1°C and continuous stirring at 50 r/min.^31^ The patches (3×3 cm) were floated in dissolution medium and 2ml of medium was extracted at different time intervals. Finally, Total phenol of each sample was analyzed and the rate of release was determined by modeling of the release pattern using regression with suitable R^2^.

### Statistical design

A box behnken method was applied to design, analyze, and optimize the myrtle patches. The concentration of gelatin, pectin, PVP, and methyl cellulose was selected as independent variables (Table 1). Accordingly, bioadhesion, patch thickness, the rate of polyphenol release, and swelling index were examined as dependent variables. Taken together, a total of 29 runs with 5 center points were designed and conducted (Table 2). The results were analyzed with model fitting based on ANOVA test, and P values< 0.05 were considered as significant.

## Results and Discussion

Different parts of myrtle are rich in polyphenolic compounds such as phenolic acids, tannins, and flavonoids explaining its antiseptic effects.^32^ Myrtle phenolic compounds are highly soluble in media resembling saliva. Therefore, the retention time of myrtle ingredients on aphthous ulcers is extremely low when is formulated in aqueous form.^33^ Considering the chronic and recurrent nature of these ulcers, mucoadhesive patches seem appropriate to increase the drug exposure time and, hence, treatment efficiency.

In this investigation, a box behnken design was applied to develop an efficient mucoadhesive myrtle patch. As summarized in Table 2, the effect of several variables was studied in total of 29 formulations with different polymer combinations. At first, screening was performed to find the most critical factors. In this step, the concentration of myrtle, as active ingredient, and PG, as plasticizer, were considered as constant. The screening outcomes clarified methyl cellulose as the cornerstone polymer. Therefore, the ratio of other polymers was determined based on this polymer.

### Thickness of the patch

**Figure 1:**
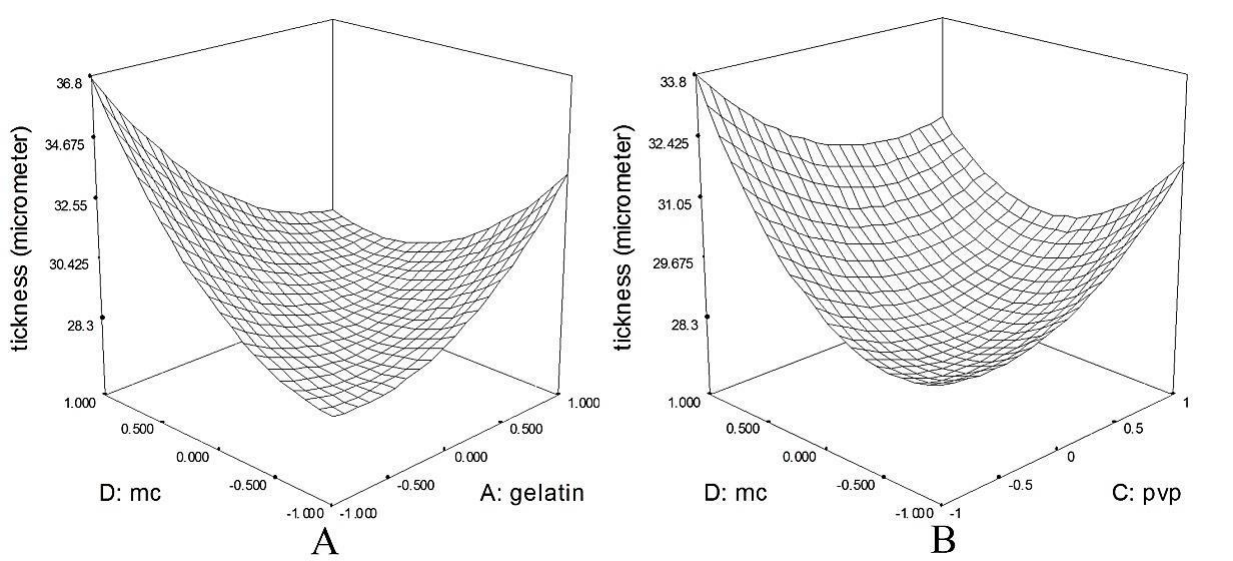
The interaction between Methyl cellulose and gelatin (A) and Methyl cellulose and Polyvinyl pyrrolidone (B) on thickness of patch. acording to figure, the minimum thickness can obtain with combination of gelatin or Polyvinyl pyrrolidone with the ratio of 1:1.

the prepared films were uniform in thickness with smooth surface. The films thickness in different formulations was in the range of 27.60 to 38.30 μm (Table 3). Furthermore, a modified quadratic model with the following equation was fitted on the data (p: 0.0004); thickness of ptach =+28.58-0.56*A+0.73*B+0.11*C+1.07*D-3.07*A*D-1.30*C*D+1.58*A2+1.11*C2+1.84*D2; where, A, B, C, and D are gela\

in, pectin, PVP, and MC, respectively. Indeed, MC (β: +1.07, p: 0.014) was the main determining factore for the patch thickness. That is, the more the ratio of MC, the thicker the final patch. Also, there were a significant interaction between MC and Gelatin (p: 0.0003). In this regard, the minimum thickness was obtained with combination of gelatin or PVP with the ratio of 1:1 (Figure 1). The thickness of film is essential factor in interaction of film with biological and patients compliance. As it was shown, the film thicness is affected by methyl cellullse as a film former agent. In parallel, Esmaeili A et al, showed that the thickness of methyl cellulose film is dependent on the ratio of film former and other additives.^34^ Also it can be affected by the interaction of other polymers with MC in the film.^35^

**Table 2:** Design of experiment in case of 29 runs according to box-behnken design

Runs	Gelatin	Pectin	polyvinyl pyrrolidone	Methylcellulose
1	1	1	0	0
2	1	0	0	1
3	0	-1	1	0
4	0	0	0	0
5	1	0	0	-1
6	-1	0	0	1
7	0	0	0	0
8	1	0	-1	0
9	0	0	0	0
10	1	-1	0	0
11	0	0	-1	1
12	0	-1	0	1
13	-1	0	0	-1
14	1	0	1	0
15	0	-1	0	-1
16	0	1	-1	0
17	-1	1	0	0
18	0	0	0	0
19	0	1	0	-1
20	0	0	1	1
21	0	-1	-1	0
22	-1	-1	0	0
23	0	0	0	0
24	0	0	1	-1
25	-1	0	-1	0
26	-1	0	1	0
27	0	1	1	0
28	0	1	0	1
29	0	0	-1	-1

**Table 3:** The magnitude of various responses for all formulations

	**Mucoadhesiveness** **(N/cm** ^ 2 ^ )	**Release rate** **(min**^-1^)	**Swelling ratio** **(%)**	**Thickness** **(µm)**	**Tensile strength** **(N/cm)**	**Disintegration** **time**
**F1**	125	37.87	222.8	30.3	295	10h
**F2**	75	39.04	256.8	31	225	9h
**F3**	200	35.8	209.9	29.3	400	11h
**F4**	140	37.26	189.3	29	355	10h
**F5**	130	38.07	276.6	33.3	215	4h
**F6**	160	35.33	136.4	38.3	695	>24h
**F7**	120	35.51	238	30.3	345	10h
**F8**	110	27.5	260.3	29.6	230	3h
**F9**	140	37.22	240.7	29.3	500	>24h
**F10**	140	33.44	272.6	28.6	345	10h
**F11**	100	33.57	259.7	35.3	550	>24h
**F12**	120	36.35	175	30	200	9h
**F13**	135	36.3	167.6	28.3	695	8h
**F14**	120	39.26	206.4	30.7	360	3h
**F15**	180	38.92	215	28.3	170	3h
**F16**	175	36	214.9	29	435	8h
**F17**	190	36.92	192.7	31.3	755	>24h
**F18**	130	38.37	205	28	355	10h
**F19**	125	37.96	198.4	30.3	445	9h
**F20**	135	39.38	195.5	30	460	6h
**F21**	210	35	195.6	29	335	6h
**F22**	165	41.17	131.9	29.6	705	11h
**F23**	180	38.16	220.8	27.6	610	6h
**F24**	150	39.88	316.9	30.6	660	6h
**F25**	180	36.45	192.4	30.7	605	12h
**F26**	210	38.84	208	32	560	10h
**F27**	155	37.37	229.4	33	715	9h
**F28**	90	39.52	241.3	29.7	1175	6h
**F29**	195	29.1	210.6	30.7	580	4h

### Tensile strength

**Figure 2:**
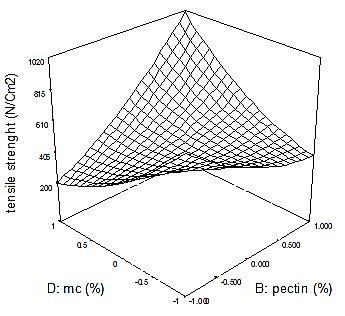
The interaction between Methyl cellulose and pectin on tensile of patch. according tothe Figure the interaction of Methyl cellulose and pectin content in formulation resulted in dramatic increase in patch tensile strength.

As summarized in Table 3, the type of polymer played a crucial role in film resistance against breaking apart. A modified quadratic model with the following equation was fitted to the tensile strength data; tensile strenght =+424.57-195.42*A+93.58*B+35.00*C-0.17*D+53.75*BC+310.50 * BD-42.50 * CD+85.92 * B^2^+97.79 * D^2^; where, A, B, C, and D are gelatin, pectin, PVP, and MC, respectively. Indeed, gelatin with negative and pectin with positive coefficients significantly influenced the patch consistency. Furthermore, augmentation of MC with pectin resulted in dramatic increase in tensile strength (Figure 2). The data were in agreement with El Halal SL^36^ and Dogan N^37^ studies that showed the addition of cellulose resulted in increase in total strength of edible film. Also pectin is known as a rigid polymer^38^ and need to modifications with different material to use in the films. This indicates that polysaccharides play a pivotal role in tensile endurance. In this regard, the hydroxyl groups in the structure of polysaccharides provide the possibility of hydrogen bond formation.^39^ Accordingly, polymer chain cross linking as well as polymer interaction with myrtle ingredients results in a stronger matrix.^40^ In addition, pectin, a complex polysaccharide, produces hydrogen and non-covalent bonds with cellulose, and acts as a binder. Therefore, pectin and cellulose synergistically increase the tensile strength. This explains why formulations containing these excipients exhibited maximal endurance against physical tension.^41^

Data analysis showed that there was no correlation between the tensile strength and thickness of patches. It showed that the containing polymer and polymer chain interactions determined the tensile strength of patch and polymer amount has minimal effect on this property.

### Swelling ratio and surface pH 

Polymers with pK equal to that of extract helped develop films with narrow range of neutral surface pH (7-7.4) in all formulations that is suitable for oral ulcer which is sensitive to extreme acidic or basic condition and also it is suitable for oral application without mucosal irritation.^42^

Swelling behavior plays a pivotal role in the pattern of drug release as well as the mucoadhesiveness of patch.^43^ It depends on several factors such as the physicochemical properties of the ingredients. Indeed, water solubility and wet ability of polymers determines the ability of water absorption. Accordingly, matrix integrity and disintegration rate defines the final extension of patch.^43^ This indicates that the type and ratio of polymers determine the swelling index and, subsequently, the rate and pattern of drug release and bio-adhesiveness.

**Figure 3:**
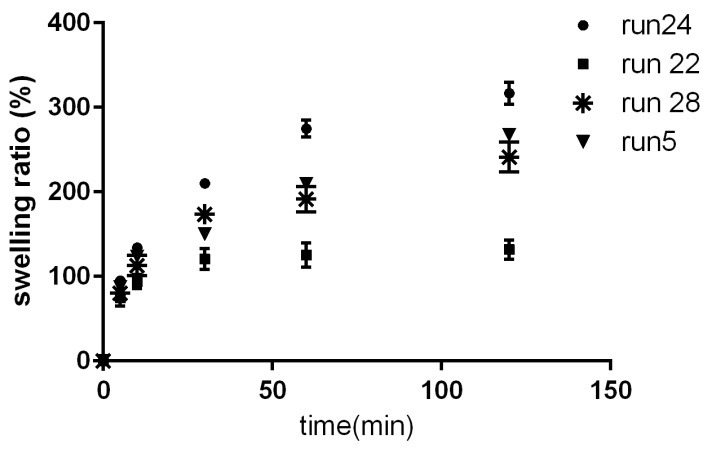
The swelling pattern of selected formulations (run 5 : 31.3% gelatin, 18.8 % pectin, 18.8% polyvinyl pyrrolidone, 31.3 % Methyl cellulose; run 22 : 1.4 % gelatin, 7 % pectin, 21.1 % polyvinyl pyrrolidone, 70.5 % Methyl cellulose; run 24 : 16.7 % gelatin, 19.2 % pectin, 32.1 % polyvinyl pyrrolidone, 32.1 % Methyl cellulose; run 28 10.2 % gelatin, 19.5 % pectin, 11.7 % polyvinyl pyrrolidone, 58.6 % Methyl cellulose).

**Figure 4:**
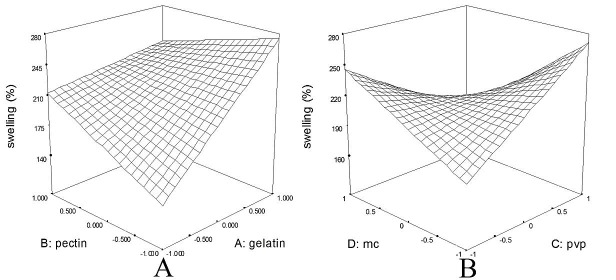
The interaction between pectin and gelatin (A) and Methyl cellulose and Polyvinyl pyrrolidone (B) on swelling of patch. According to figure, the maximum swelling index is approachable with maximum ratio of gelatin vs minimum ratio of pectin in formulation. On the other hand, there is the lowest swelling ratio when Polyvinyl pyrrolidone and Methyl cellulose are employed equally in the formuation.

The swelling ratio in formulations varied from 131.90% to 316.90%.As such, different formulations exhibitted distinct patterns of swelling (Figure 3). Overal, a 2FI vs Linear model was fitted on the data (p: 0.0002); Swelling index =+216.57+38.88*A+8.29*B+2.72*C-10.03*D-27.65*A*B-42.63*C*D; where, A, B, C, and D are gelatin, pectin, PVP, and MC, respectively. This indicates that gelatin (β: +38.88) was the most crucial factor for swelling compared to other polymers. That is, the more the ratio of gelatin in formulation, the higher the swelling index of the resulted patch. Also, there was an interaction between glatin and pectin (p:0.0439). also there was an interaction between MC and PVP (p: 0.0033). In particular, maximum swelling index was observed in formulations containing maximum ratio of gelatin and minimum ratio of pectin. On the other hand, formulations containing equal amounts of PVP and MC exhibitted the lowest swelling index (Figure 4).

Hydrophilic groups such as –OH, -COOH and –NH_2_ in gelatin structure provides the possibility of hydrogen bond formation, and thereby high water absorption. This explains the positive impact of gelatin on swelling index. In this regard, the swelling index of gelatin depends on ionization of the functional groups. Therefore, the medium pH as well as electrolyte concentration and presence of other complexing agents influence the ionization state of gelatin.^44^ This highlights the importance of other factors in gelatin-induced alteration of swelling index. In fact, formulations containing pectin and gelatin demonstrated lower swelling ratio compared to those containing only gelatin. This may be explained by the fact that pectin, with lots of -COOH group, interferes with –NH_2_ groups in gelatin, thereby, decreases the capacity of gelatin for hydrogen bond formation and water absorption. In this regard, Mishara et al showed that increasing the ratio of gelatin in the pectin film enhanced the swelling index by improving the film porosity.^44^

PVP, by itself, didn’t have any significant effect on swelling index. However, it did decrease the swelling ratio of patches containing MC (β: -42.63). This seems contrary to the high aqueous solubility of PVP which can enhance the wet ability and water absorption and disintegration rate of the hydrophilic matrix. In this regard, PVP enhanced the swelling of chitosan films, an insoluble polymer.^43^ whereas no portion of highly soluble polymers such as HPMC lead to increasing the swelling of patch.^45^

### Mucoadhesiveness test

We observed that gelatin (β: -25), MC (β: -19), and pectin (β: -12) were main factor influencing mucoadhesiveness of the patches. The result showed that the mucoadhesiveness of the myrtle patches was acceptable when polymers are used in low concentration. One of the most important factors affecting mucoadhesion is the concentration of polymers. Higher level of polymers is responsible for forming highly coiled structure of the polymers which can reduce the polymer chains flexibility and interaction with mucin. Subsequently, the mucoadhesion force will fall under acceptable value.^46^ Namely, Malik and his coworkers indicated that increasing the concentration of chitosan in the ondansetron loaded beads will decrease the mucoadhesion force.^47^ In this regard, the data best fitted to the following equation; mucoadhesiveness =+138.24-25.00*A-12.92*B+3.33*C-19.58*D- 20.00*A*D+20.00*C*D+17.18*B^2^+20.30*C^2^–17.82*D^2^; where, A, B, C, and D are gelatin, pectin, PVP, and MC, respectively. This indicates a synergistic effect between PVP and MC (β: +20).

Mucin in the structure of buccal surface is negatively charged. Therefore, positively charged excipients in formulation ought to enhance bioadhesion. In particular, high molecular weight polymers such as pectin as well as insoluble ones like chitosan produce stronger interactions with mucin.^48^ However, we found that pectin had a negative impact on bioadhesion (β: -12). As such, positively charged PVP did not significantly alter this parameter (β: +3.33, p> 0.05). This observation is supported by Jiyeon and coworkers. They designed a bilayer mucoadhesive strip of lidocaine and showed that PVP was not able to enhance mucoadhesivity. However, they showed that addition of HPMC to the bilayer strips increased the mucoadhesion strength.^49^ This indicates that bioadhesion is not determined only with surface charge density. In fact, several factors come to play including surface charge density, flexibility, polymer molecular weight, swelling rate, type of the biological surface to which the patch is adhered and the adherence time.^43^ For instance, polymers enhancing the swelling index seem to reduce bioadhesion. In this regard, gelatin (β: +38.88) and MC (β: +10.03) significantly enhanced swelling index. In contrast, they had an inverse effect on bioadhesion (β: -25 and -19.58 for gelatin and MC, respectively). In this regard, patches produced with chitosan matrix having gelatin displayed less mucoadhesive strength compared to those having only chitosan.^50^

### Myrtle release of patch

According to Table 3, almost the formulations composed the myrthys with long perod of disintegration time up to more than 24 houre in some formulations. Howevere polyphenolic compound was released of formulation between 3 to 6 houres.

Overal, the release rate best fitted to the following model; release rate =+37.49-0.82*A+0.41*B+2.74*C+2.17*A*B+2.34*A*C-1.82*C^2^; where, A, B, C, and D are gelatin, pectin, PVP, and MC, respectively. This indicates PVP as the main factor determining polyphenol release from the patch. In fact, there was a positive correlation between the ratio of this cationic polymer and the rate of myrtle release from the patch (β: +2.74, F-value: 23.68).

Polyphenols are among water soluble materials. Therefore, their release from the matrix ought to have a direct relationship with the capacity of patch to absorb water. In this regard, water soluble polymers such as PVP enhance water absorption, promotes the drug release and patch dissolution. In addition, PVP as a pore-former polymer creates lots of water channels, and cause perturbation of the matrix consistency. Subsequently, these channels allow better water penetration, swelling and ultimately faster drug diffusion.^51^ Similarly, PVP is shown to enhance the release of soluble drugs such as sumatriptan succinate and felodipine from polymeric matrix.^52,53^

**Figure 5:**
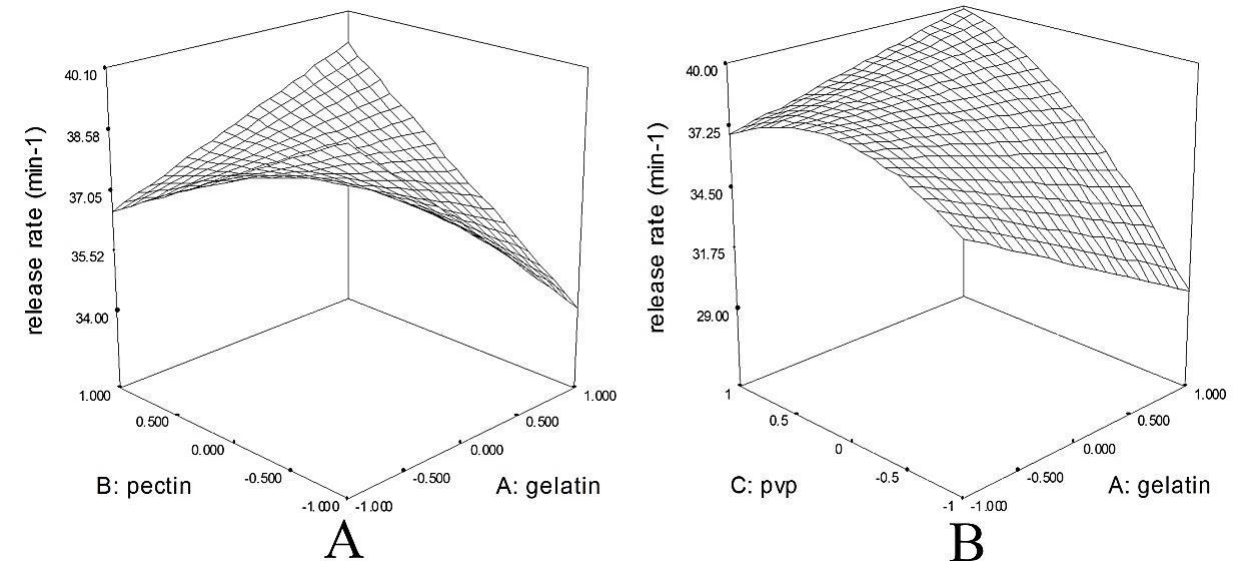
The interaction between Polyvinyl pyrrolidone and gelatin (A) and pectin and gelatin (B) on release myrtle from patch. According to the Figure, the correlation between the weight of patch and drug release. It shows that the amount of solid content in the patch is another factor that can delay the extract release.

In contrast, other polymers did not influence polyphenol release by themselves, although their effect was significant when used in combination. In this regard, co-formulation of gelatin and pectin (β: +2.17) as well as gelatin and PVP (β: +2.34) enhanced polyphenol release from the patch. Similarly, it was observed that gelatin concentration solely, does not play a crucial role in release of bupivacaine.^54^ The failure of gelatin and MC, highly water soluble polymers, to influence drug release may seem contrary to the above mentioned direct relationship between the water solubility of polymer and drug release. This suggests a potential role for other factors. For instance, the composition of patch may influence the disintegration rate via mechanical properties of sol-gel interface with water.^55^ In addition, total patch weight influenced the release rate (Figure 5). As such, solid content in the patch is another factor that can delay the extract release.^56^ In fact, higher solid content diminishes matrix porosity, thereby inhibiting water penetration and outward movement of drug from matrix.^55^ In this regard, increase in pectin and gelatin concentration delayed polyphenol release from the matrix (Figure 5A). Similarly, Ikram and coworkers showed that the release of drug from the matrix is dependent on the polymer properties and specially the viscosity of the polymeric matrix after water diffusion inside. Also the immobile water in the matrix by increasing the swelling index, create the new condition for drug release. All together the total tendency of polymer to water absorption and the ratio of patch swelling as well as polymer viscosity will identify the fate of drug release.^57^ So the higher content of gelatin in combination with PVP or pectin in the myrtle patches, with high water absorption and swelling index, leads to increase the rate of drug release. However, optimization of the ingredients is necessary to obtain the patch with desire release rate.

### Optimization

According to the optimal value for each response, the best formulation was predetermined. In fact, the ratio of each excipient was predicted to result in the desired response. Then, the final formulation was determined to get a patch with optimal values in all responses (Table 4). The calculated optimal formulation was composed of PVP, MC, pectin, gelatin and myrtle extract with ratios of 1:13:1:5:3, respectively.

**Table 4:** Predicted value of optimum formulation

	**Gelatin**	**Pectin**	**Polyvinyl** **pyrrolidone**	**Methylcellulose**	**Release rate** **(min**^-1^)	**Swelling ratio** **(%)**	**Thickness** **(µm)**	**Desirability**
Predicted value	25	5	5	65	27.5	291.9	30.0	0.92

The final patch was produced with the predicted ratios for optimal formulation. Then, dependent variables were studied once again for the final patch. The results demonstrated good agreement between the predicted and observed responses.

## Conclusion

The suitable oral patch of *myrtus communis* L. can develop with the aid of soluble polymer including polyvinyl pyrrolidone, gelatin, pectin and methyl cellulose. Inclusion of gelatin in myrtus patches helps to higher swelling and hydration but with negative effect on mucuadhesive property. Pectin same as gelatin compose of oral patch with higher tensile strange. The release of myrtus extract is depended on water solubility of polymer. And, PVP as a low molecular water soluble polymer make the extract release faster. Altogether, optimization of the hydrophilic polymer in the patch, made it so flexible with degradation time more than 24 h and release rate of 27.5 (min^-1^) and swelling ratio of about 300%.

## Acknowledgments

This research was granted by research department of Shahid Sadoughi University of Medical Science and Health Services. This research was based on a thesis submitted for Pharm. D degree (No. 3602).

## Ethical Issues

Not applicable.

## Conflict of Interest

The authors declare no conflict of interests.
